# Improved simultaneous LET and dose measurements in proton therapy

**DOI:** 10.1038/s41598-022-10575-4

**Published:** 2022-05-18

**Authors:** Jeppe Brage Christensen, Michele Togno, Lily Bossin, Oskari Ville Pakari, Sairos Safai, Eduardo Gardenali Yukihara

**Affiliations:** 1 Department of Radiation Safety and Security, Paul Scherrer Institute, Villigen PSI, Switzerland; 2 Center for Proton Therapy, Paul Scherrer Institute, Villigen PSI, Switzerland

**Keywords:** Radiotherapy, Optical techniques, Materials for devices

## Abstract

The objective of this study was to improve the precision of linear energy transfer (LET) measurements using $\text {Al}_2\text {O}_3\text {:C}$ optically stimulated luminescence detectors (OSLDs) in proton beams, and, with that, improve OSL dosimetry by correcting the readout for the LET-dependent ionization quenching. The OSLDs were irradiated in spot-scanning proton beams at different doses for fluence-averaged LET values in the (0.4–6.5) $\hbox {keV}\, \upmu \hbox {m}^{-1}$ range (in water). A commercial automated OSL reader with a built-in beta source was used for the readouts, which enabled a reference irradiation and readout of each OSLD to establish individual corrections. Pulsed OSL was used to separately measure the blue (*F*-center) and UV ($F^+$-center) emission bands of $\text {Al}_2\text {O}_3\text {:C}$ and the ratio between them (UV/blue signal) was used for the LET measurements. The average deviation between the simulated and measured LET values along the central beam axis amounts to 5.5% if both the dose and LET are varied, but the average deviation is reduced to 3.5% if the OSLDs are irradiated with the same doses. With the measurement procedure and automated equipment used here, the variation in the signals used for LET estimates and quenching-corrections is reduced from 0.9 to 0.6%. The quenching-corrected OSLD doses are in agreement with ionization chamber measurements within the uncertainties. The automated OSLD corrections are demonstrated to improve the LET estimates and the ionization quenching-corrections in proton dosimetry for a clinically relevant energy range up to 230 MeV. It is also for the first time demonstrated how the LET can be estimated for different doses.

## Introduction

The proton therapy community increasingly focuses on the relative biological effectiveness (RBE) and the effect of the linear energy transfer (LET) distributions in treatment plans^1^. Several detectors, such as semiconductors^2^, gas counters^3^, and radiochromic films^4^, have been proposed to measure the LET of hadrons at conventional dose-rates. Nevertheless, dosimetry in hadron beams remains challenging because of the typical under-response due to the ionization quenching of otherwise dose-rate independent detectors^5–7^.

Ionization quenching is occasionally exploited to estimate the LET by comparing the response of two detectors having different quenching characteristics, e.g. scintillators relative to absorbed dose calorimeters^8^ or scintillators relative to ionization chambers^9^. Similarly, pairs of differently quenching organic scintillators^10^, thermoluminescent detectors^11^ or phosphor films^12^ have been used to simultaneously estimate dose and LET, which, however, is associated with large uncertainties in regions with steep gradients, e.g. at the distal edge.

The optically stimulated luminescence (OSL) of $\text {Al}_2\text {O}_3\text {:C}$ allows the estimation of both dose and LET, but with the advantage of only requiring a single detector, and has been demonstrated^13^ to be dose-rate independent in proton beams up to ${150}\,\hbox {kGy}\,\hbox {s}^{-1}$.

$\text {Al}_2\text {O}_3\text {:C}$ OSL is associated^14^ with a fast UV emission (centered at 335 nm, lifetime $< {7}\,\hbox {ns}$) and a much slower blue emission (centered at 420 nm, ${35}\,\hbox {ms}$ lifetime). The emission in the blue band is traditionally used for dosimetry with a negligible time dependence after irradiation^14^. The emission in the UV band is less favorable for dosimetry, but it has been demonstrated that the ratio of the two emission bands, measured using pulsed OSL (POSL), can be used to establish an LET calibration curve and estimate the LET in proton beams^15–18^, and heavier ions^7^. However, previous proton LET studies using $\text {Al}_2\text {O}_3\text {:C}$ focused on LET estimations for constant doses around ${0.2}\,\hbox {Gy}$ and LETs below the elevated LET at the distal edge^19,20^.

Previous studies on the possibility of LET estimation using OSL relied on custom-build readers designed to achieve the type of specialized POSL measurements required for this finality^7,15^. More recently, automated OSL readers capable of POSL measurements, which offers more controlled and stable readout than previous readers, became available^21^. These readers allows for the automated irradiation of each OSLD with a reference dose using a built-in beta source. These reference irradiations enable intra-sample corrections, which have been demonstrated to greatly improve the dose estimations in photon beams^22^. Nevertheless, the possibility of improving the LET estimations using such modern readers have not been investigated yet.

This work aims at improving the precision of LET measurements using $\text {Al}_2\text {O}_3\text {:C}$ OSLDs in proton beams by taking advantage of modern, automated readers capable of POSL measurements and irradiations. Improved LET determinations will in turn improve OSL dosimetry in proton therapy, as the LET can be applied to correct the readout for the LET-dependent ionization quenching.

## Methodology

### $\text {Al}_2\text {O}_3\text {:C}$ OSL detectors

All $\text {Al}_2\text {O}_3\text {:C}$ OSLDs were prepared from a film consisting of a ${47}\,\upmu \hbox {m}$-thick layer of $\text {Al}_2\text {O}_3\text {:C}$ powder (${15}\,\upmu \hbox {m}$ median diameter) mixed with a binder and deposited on a ${75}\,\upmu \hbox {m}$-thick plastic substrate^23^. The OSLDs were cut to 7 mm diameter discs from the film and bleached prior to usage with a green LED (525 nm). The OSLDs were irradiated in packages containing five OSLDs, where each package was wrapped in opaque tape to protect the OSLDs from light exposure after irradiation. A data point is here reported as the mean of the OSL signals from the five OSLDs in each package along with the standard deviation of the data (coverage factor $k = 1$).

### Proton irradiations

All OSLD packages were irradiated at the Center for Proton Therapy of the Paul Scherrer Institute. The four ${10}\,\hbox {cm} \times {10}\,\hbox {cm}$ irradiation fields used for this study consisted of up to ($41 \times 41$) spots per energy layer and are defined in Table 1. The OSLDs were irradiated along the central beam axis of each field to investigate the effect of different doses and LET distributions. The depth was varied using PMMA slabs and the OSLD package was in each case placed next to an ionization chamber (Advanced Markus TM 34045, PTW Freiburg, Germany). The field denoted $d_1$ was used for the OSLD dose calibration.

The possible misalignment of the OSLD package relative to the ionization chamber along the beam axis was estimated to be 0.5 mm, which will be used to assess the sensitivity of the LET simulations given such a misalignment. For the ionization chamber, the type A uncertainty is estimated to be $<{0.3}\%$ $(k = 1)$. The type B uncertainty is omitted for the ionization chamber as all OSLD calibrations and dose measurements were acquired with the same chamber. The accuracy of the proton beam energy is better than 1% for all energies.

**Table 1:** The four proton fields used to irradiate the OSLDs at different LETs and doses.

Field	Energies (MeV)	OSLD positions (cm)	SOBP width (cm)	Dose (Gy)	f-$\hbox {LET}_\text {W}$ ($\hbox {keV}\, \upmu \hbox {m}^{-1}$)
$d_1$	230	2.0	–	0.1–1.1	0.44
$f_1$	70	1.0–3.8	–	0.20–1.0	1.1–9.1
$f_2$	73–112	2.0–9.0	4.0	0.60–1.0	0.86–6.1
$f_3$	120–177	5.0–19	8.5	0.61–1.0	0.63–5.6

The field $d_1$ was used for the dose calibration at a depth of 2 cm PMMA. For the remaining three fields, OSLD packages were irradiated at different positions along the central axis at ranges given as the column with OSLD positions. $f_1$ was a 70 MeV single energy layer whereas $f_2$ and $f_3$ were spread-out Bragg peaks (SOBPs). The column with OSLD positions denote the depths in PMMA at which the OSLDs were irradiated. The uncertainty estimates are given in the “Proton irradiations” section.

### Automated OSL reader and corrections

The OSLDs were read out using a Risø Reader (TL/OSL-DA-20, DTU Nutech, Denmark), green light for stimulation (525 nm), and a photomultiplier tube (PMT; PMD9107Q-AP-TTL, ET Enterprises, UK) for detection. UV band-pass filters (7.5 mm total thickness, Hoya U-340, Hoya Corporation) were used to block the stimulation light from reaching the PMT.

The OSLDs were placed on stainless steel measurement discs and read in a pulsed stimulation mode^21^ consisting of alternating ${100}\,\upmu \hbox {s}$ stimulation pulses and ${100}\,\upmu \hbox {s}$ intervals without stimulation for a total measurement time of ${300}\,\hbox {s}$. The data was binned into ${3}\,\hbox {s}$ channels giving a total of 100 data points for both *on* and *off* stimulation intervals. The signal during the *off* stimulation periods was attributed to the blue emission, whereas the difference between the signal in the *on* and *off* periods was attributed to the UV emission band^14^. For each measurement the background estimated by the last 10 data points was subtracted. The integral of the OSL curve after background subtraction is here denoted *S*. Two other quantities were calculated from the data, namely the UV/blue ratio of the emission bands and the *total* emission, denoting the sum of the UV and blue emission bands during stimulation. The use of the total emission as a quenching-free way of estimating the dose is investigated below.

Individual OSLD correction factors for the blue and UV emission bands were obtained using the built-in $^{90}\text {Sr}/^{90}\text {Y}$ beta source. Following the first readout of the proton-irradiated OSLD, the OSLD was irradiated for ${30}\,\hbox {s}$, estimated from the dose calibration to correspond to about ${1.4}\,\hbox {Gy}$. The OSLD was then read out again using the same pulsing scheme, resulting in the reference integral signal $S_R$ for both emission bands.

Given the OSL signal *S* from the proton irradiation and reference signal $S_R$ from the beta source irradiation, the quantity $S/S_R$ denotes the relative intensity of the blue or UV emission band for that particular proton irradiation. Hence, the normalization of the integral OSL signal eliminates signal variations due to size or material differences^22^. The best estimate of the response of a point of measurement is calculated as the mean of the $S/S_R$ signals for all OSLDs in the package. Results presented merely as *S* denotes the average value of the OSLDs in a package without correction relative to the reference signal $S_R$. Unless otherwise specified, the results presented here are reference-corrected OSL signals, $S/S_R$.

Examples of the UV and blue OSL signals for three different LETs are shown in Supplementary Fig. A1, showing the relative increase of the UV emission with increasing LET. The OSLDs were read out between 14 and 16 days after irradiation to limit the variation of the UV emission between OSLD packages. Further details are given in Supplementary Materials, Sect. A.2.

### Monte Carlo simulations

The Monte Carlo tool TOPAS^24^ was used to simulate the dose and LET distributions along the central-beam axes of the proton fields where the OSLDs were placed. The irradiation log files generated by the beam delivery system were used to create TOPAS files containing the position and weight of each spot. Although numerous LET scoring methods are available in the literature for radiobiological studies^25^, all LET values presented here are given as the fluence-averaged LET in water (f-$\hbox {LET}_\text {W}$); similar results are achievable using dose-averaged LET. The uncertainty in the simulated LET distributions is assessed by shifting the point of measurement 0.5 mm to either side to mimic an upper estimate of a misalignment. A summary of the simulation parameters are given in Table 2 as recommended by the AAPM TG-268^26^.

The transport medium of the simulations is PMMA to reflect that PMMA slabs are used to vary the OSLD position along the central beam axes. However, the Monte Carlo-scored quantities dose and LET are scored in water in line with previous studies^17,18^.

The OSLDs are omitted from the simulations for two reasons. Firstly, since a study^9^ showed that the signal averaging over 1 mm wide plastic detectors was $<{1}\%$ in proton beams, the signal averaging over the ${47}\,\upmu \hbox {m}$ $\text {Al}_2\text {O}_3\text {:C}$ powder layer is expected to be negligible. Secondly, the OSLDs are calibrated in terms of dose and LET in water.

**Table 2:** Summary of the Monte Carlo simulation parameters used to calculate the dose and LET distributions in the proton fields.

Item name	Description	References
Code, version	TOPAS, version 3.6.1	^24^
Validation	Depth-dose measurements against simulations	^27^
Timing	12 cores, CPU time of the order of $5 \times 10^{5}\,\hbox {s}$	
Geometry	($1 \times 1 \times 1$) $\hbox {m}^3$ G4_PLEXIGLASS phantom with the surface 48 cm from the source	^27^
Source	${10}\,\hbox {cm} \times {10}\,\hbox {cm}$ energy layers with up to $41 \times 41$ unique spots. Spot positions and weights were extracted from the irradiation log files generated by the beam delivery system.	^27^
Physics	The Default TOPAS physics list	^24^
Scoring	Dose to water using the DoseToWater scorer and fluence-averaged LET using the ProtonLET scorer weighted by Track	^24^
$\#$ Histories	$10^8$ primaries for each simulation	

The number of energy layers varied with the proton field.

### LET calibration

An LET calibration curve was established by irradiating multiple OSLDs at positions with different doses and LET: the UV/blue emission ratio was for each OSLD related to the simulated LET at its position in the proton field. The shape of the UV/blue ratio distribution as a function of the LET depends on whether the OSLD corrections were applied ($S/S_R$) or not (*S*). The OSLDs were LET calibrated by fitting one of the two empirical functions

$$\begin{aligned} {f(x) = } {\left\{ \begin{array}{ll} a_1\left( 1- \exp (-a_2 \, x) \right) + a_3,\,\,\,\,\,\,\,\,\,\,\,\,\,\,{\text(1a)}\\ a_2 + \dfrac{a_1 - a_2}{1 + a_3 \, x^{a_4}}. \,\,\,\,\,\,\,\,\,\,\,\,\,\,\,\,\,\,\,\,\,\,\,\,\,\,\,\,\,\,\,\,\,\,\,\,\,\,{\text(1b)}\end{array}\right. } \end{aligned}$$

to the data, where $x=\text {LET}$ and $a_i$ (for $i = 1,\ldots ,4$) are coefficients to be determined. Equation (1a) represents a saturating exponential function which was found to better represent the data here than a quadratic function used in a previous study^16^, whereas Eq. (1b) was found suitable for quenching corrections for heavier ions^7^. The LET calibration curve obtained using the UV/blue emission ratio without reference corrections (*S*) was found to better represent the data using Eq. (1a), whereas the reference corrected UV/Blue ratio ($S/S_R$) was fitted to the LET using Eq. (1b). All fits were acquired with the LMFIT package^28^ in python3.9 using the default options.

### Dose calibration

The OSLDs were dose calibrated using a 230 MeV proton beam against the same ionization chamber used to measure the proton fields. In each case, a package containing OSLDs was placed below 2 cm PMMA next to the ionization chamber and irradiated in a ${10}\,\hbox {cm} \times {10}\,\hbox {cm}$ field. The OSLD packages and ionization chamber were irradiated with doses in the $(0.1{-}1.1)\,\hbox {Gy}$ range to cover the range relevant to the proton fields in Table 1. The saturating exponential in Eq. (1a) was fitted to the $S/S_R$ OSLD response as a function of the dose measured with the ionization chamber in line with previous works^13^.

### Ionization quenching corrections

To quantify the energy dependent (ionization quenched) response of the OSLDs, the relative luminescence efficiency is defined as

2 $$\begin{aligned} \eta = \dfrac{D_\text {OSLD,water}}{D_\text {water}}, \end{aligned}$$

where $D_\text {water}$ denotes the absorbed dose to water and $D_\text {OSLD,water}$ is the dose determined by the $\text {Al}_2\text {O}_3\text {:C}$ OSLD, cross-calibrated in absorbed dose to water using the ionization chamber in the 230 MeV proton beam. Thus, given the dose calibration at 230 MeV, the relative luminescence efficiency is $\eta = 1$ for a 230 MeV proton and decreases non-linearly with the proton energy due to the ionization quenching.

For a given OSLD readout, the intensity of the blue emission is thus related to the quenched dose via the dose calibration obtained as described in the “Dose calibration” section, whilst the ratio of the UV and blue emission intensities is used to calculate the LET using the LET calibration obtained as described in the “LET calibrations” section. The relative luminescence efficiency in Eq. (2) mapped as a function of the LET enables a calculation of the (quenched-corrected) dose by scaling the quenched dose by $\eta ^{-1}$ for the given LET.

## Results and discussion

### Dose and LET distributions for the proton fields

**Figure 1:**
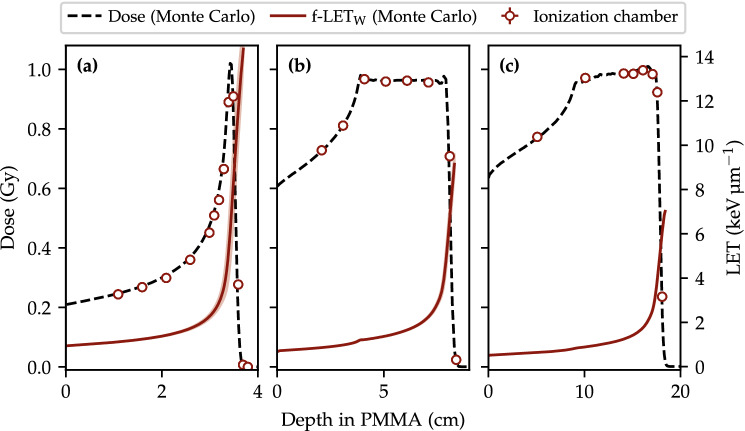
The three fields in Table 1 where the single energy layer $f_1$ is shown in (**a**), the SOBP $f_2$ in (**b**) and SOBP $f_3$ in (**c**). Open markers denote measurements with an ionization chamber placed next to the OSLDs during the irradiations. The simulated f-$\hbox {LET}_\text {W}$ depth profiles are shown with solid lines along with a band showing the LET given a 0.5 mm misalignment for reference.

Figure 1 shows the simulated dose and fluence-averaged LET along the central beam axis for the proton fields used in this study. The points of measurements with the ionization chamber (open markers) coincide with the locations of the OSLD packages. The agreement between the measured and simulated dose along the central beam axis validates the use of the Monte Carlo model to simulate the LET distribution at the positions where the OSLD packages are placed. The variation in the simulated LET for a 0.5 mm shift to either side of the point of measurement amounts to less than 1% at the SOBP plateaus and is plotted in the figure as a band around the f-$\hbox {LET}_\text {W}$.

#### Automatic OSLD corrections

**Figure 2:**
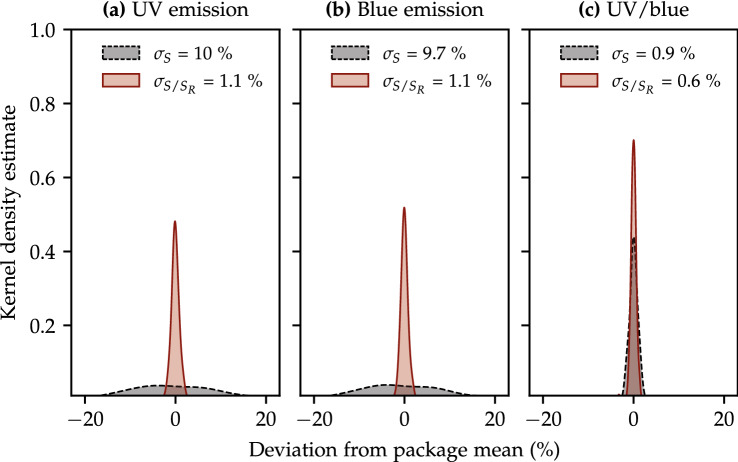
The kernel density estimates of 150 OSLDs plotted as the deviation of each OSLD from the mean of its package. The dashed black line denotes the distribution where the OSLD signal *S* with spread $\sigma _S$ is not corrected using the reference irradiation $S_R$. The red solid line denotes the reference-corrected quantity $S/S_R$ with spread $\sigma _{S/S_R}$.

The impact of the reference irradiation is investigated in Fig. 2. The figure shows the kernel density of the deviation of each OSLD from the package mean for the signal *S* and for the ratio $S/S_R$ for 150 detectors (30 packages with five $\text {Al}_2\text {O}_3\text {:C}$ OSLDs, each irradiated at different LET or dose). Results are shown for the UV (Fig. 2a) and blue (Fig. 2b) emission bands, as well as for the ratio between the two emission bands (Fig. 2c), which is used for LET measurements. The spread of the Gaussian kernel is presented in the legend of each subplot.

The spread of the uncorrected measurements ($\sigma _S$) for both the blue and UV emission bands is around 10%, improving to 1.2% after the reference correction has applied. This result is similar to that reported for photons^22^.

The uncorrected UV/blue emission ratio, which is relevant to the LET measurements, is well-defined with a spread around 0.9%. This low spread is expected, because the UV/blue ratio is calculated from signals obtained from the same OSLD and, therefore, should not be dependent on the detector size or sensitivity. It is also in agreement with a previous study^7^ and highlights the advantage of using the same detector for the LET determination.

Nonetheless, it was found that the correction based on the reference irradiation improves the measurements of the UV/blue ratio further, as evident from Fig. 2c, reducing the sample variability to 0.6%.

#### Dose calibration and detector efficiency

**Figure 3:**
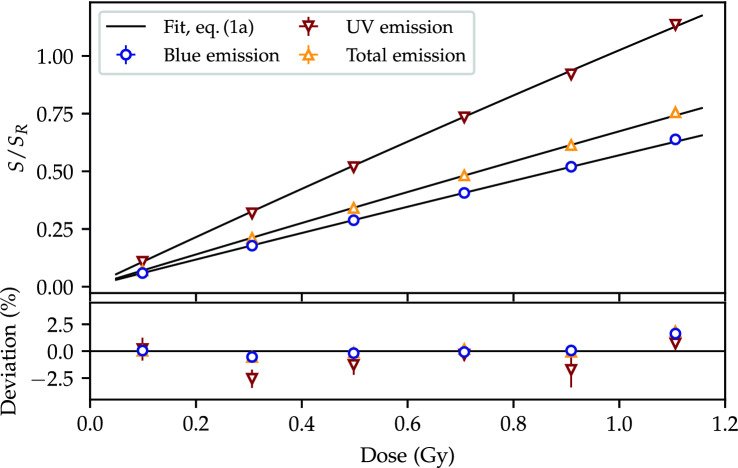
The 230 MeV dose calibration fitted with the saturating exponential in Eq. (1a) for the emission in the blue and the UV emission bands as well as the sum of the two during stimulation (denoted total emission).

The dose calibration curves for the UV and blue emission bands, as well as the sum of the two (denoted total), are shown in Fig. 3. Although the $\text {Al}_2\text {O}_3\text {:C}$ response has been reported^29^ to be linear in the $(0.1{-}1.1)\,\hbox {Gy}$ range, the use of the $S/S_R$ quantity gives a non-linear behaviour, and the dose calibration is thus fitted with the saturating exponential^22,30^ in Eq. (1a). Data points corrected by the reference irradiation show a reproducibility at a 1% level.

**Figure 4:**
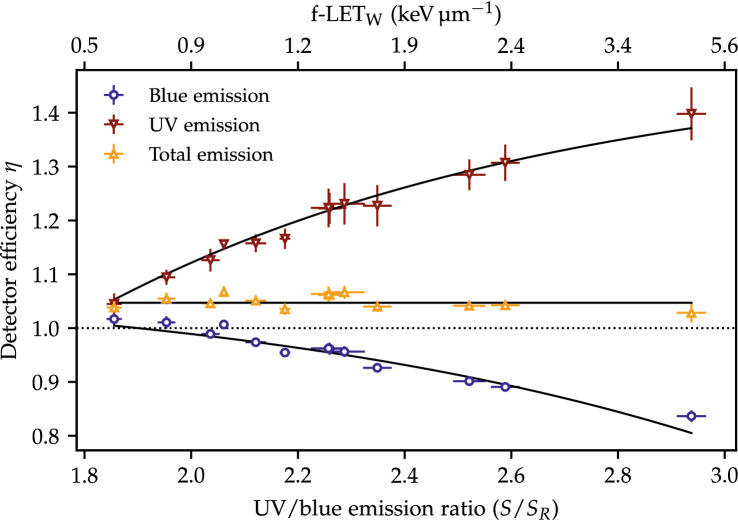
Relative luminescence efficiency for the $\text {Al}_2\text {O}_3\text {:C}$ OSLD as a function of UV/blue ratio for the three quantities used for dose calculations. The LET values at certain points are shown for reference.

The relative $\text {Al}_2\text {O}_3\text {:C}$ luminescence efficiency is shown in Fig. 4. The doses for the OSLD packages irradiated at the two SOBP plateaus, calculated with the dose calibrations in Fig. 3, are plotted relative to the ionization chamber measurements as given by Eq. (2). In contrast to the results for radiochromic film^31^, where only the dose-averaged LET could describe the relative detector efficiency for different beams, both the dose- and fluence-averaged LET are here found to correlate well with the detector efficiency for the $\text {Al}_2\text {O}_3\text {:C}$, in agreement with a previous study^16^. The OSLDs are here only calibrated against the fluence-averaged LET although similar results can be obtained using the dose-averaged LET.

The measurements taken at the single energy layer are here omitted due to the risk of misalignment at the Bragg peak region. Whilst other studies have published similar detector efficiencies^7,32,33^, a one-to-one comparison between the data sets is not appropriate due to the variations in the equipment, readout methods as well as variations in the storage time before readout.

Nonetheless, the figure shows the well-known quenching of the emission in the blue band as a function of the ionization density. The use of the UV emission band for dose measurements is difficult because of its build-up as a function of storage time [see Fig. A2(b) in the Supplementary Materials]. The relative detector efficiency as a function of the UV/blue ratio will be used to correct the quenched dose measured with the OSLDs.

#### Quenching-free OSLD dose measurements

The sum of the intensities of blue and UV emission bands during stimulation, denoted the total emission, shows an almost constant detector efficiency as a function of the LET in Fig. 4. The magnitudes of the UV and blue emission bands are greatly affected by several parameters, in particular the pulsing parameters, the absorption filters, and the PMT sensitivity. Nevertheless, the results indicate that it may be possible to engineer the experimental setup or data processing to obtain LET-independent dose measurements for $\text {Al}_2\text {O}_3\text {:C}$. This would be comparable to the OSL material $\text {MgB}_4\text {O}_7\text {:Ce,Li}$, which has been demonstrated to have an LET-independent dose response for protons^34^. Such a study is, however, not pursued further here. One should also keep in mind that the UV/blue emission ratio changes with time after irradiation [see Fig. A2 in the Supplementary Materials] and, therefore, this result may be valid only for a constant delay between irradiation and readout.

### LET calibration

**Figure 5:**
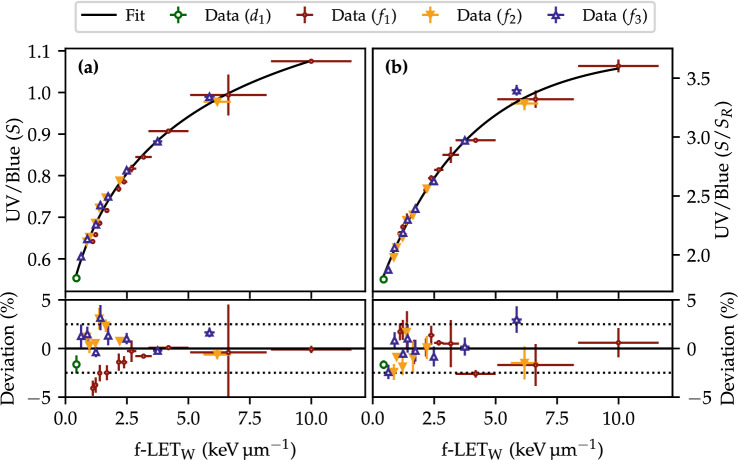
The LET calibration curves for the (**a**) UV/blue ratio without corrections and (**b**) the UV/blue ratio scaled by the UV/blue ratio from the reference irradiations. The markers and function fits are explained in the text. The legend refers to the fields defined in Table 1.

The $\text {Al}_2\text {O}_3\text {:C}$ LET calibration curve is shown in Fig. 5 for the two cases where the UV/blue ratio is either left uncorrected (Fig. 5a) or corrected relative to the reference UV/blue ratio (Fig. 5b). Four sets of data were used for the LET calibrations to acquire the OSLD response at different LETs and doses. The figure legend refers to the field names as defined in Table 1. The data in Fig. 5a is fitted with the function in Eq. (1a) whereas the saturating exponential in Eq. (1a) was found to better represent the data in Fig. 5b.

A systematic discrepancy is observed in the residuals of Fig. 5a for the data obtained in the single energy layer [‘Data ($f_1$)’]. A trend is observable where the deviation of the data increases with a decrease in LET. These data points were acquired at different doses $(0.2{-}1.0)\,\hbox {Gy}$ in contrast to the majority of the data points measured near the ${1}\,\hbox {Gy}$ SOBP plateaus. This discrepancy is partly attributed to the filling of deep traps in the crystal which is known to be dependent on both dose^35^ and LET^15^. Hence, previous studies^7,18^ acquired all data for the LET calibration at the same dose around ${0.2}\,\hbox {Gy}$.

If present at all, the systematic discrepancy due to the simultaneous variation of dose and LET in Fig. 5a is much less pronounced in the $S/S_R$ calibration curve in Fig. 5b. This indicates that the reference-corrected UV/blue ratio is less sensitive to dose variations as the variations in the deep trap filling during the proton irradiation also affects the reference UV/blue ratio. Effectively, this allows an LET calibration at different doses and, furthermore, implies a capability of measuring LET at different doses.

Along with the results of the sample variability in Fig. 2c, showing that the reference-corrections reduce the UV/blue variability, the results from Fig. 5b show that the reference-corrected ($S/S_R$) UV/blue ratio improves the overall LET estimations relative to the uncorrected UV/blue ratio.

#### LET estimations

**Figure 6:**
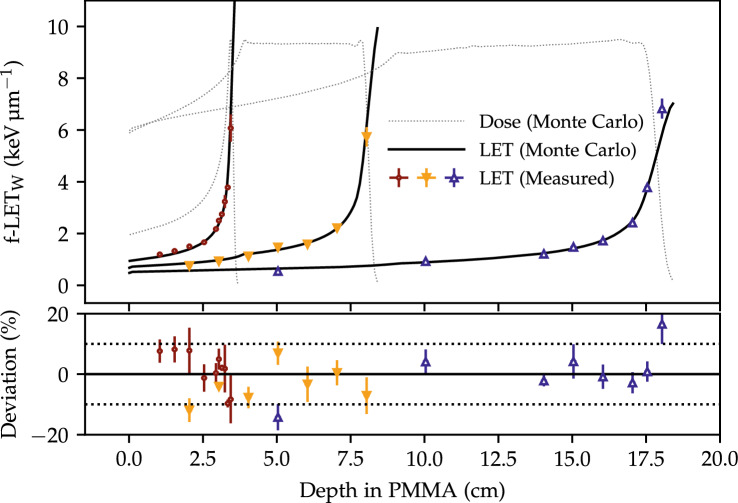
The estimated LET for the three fields $f_1$, $f_2$, and $f_3$ using the $S/S_R$ LET calibration curve along with the simulated LET (solid lines). The horizontal dashed lines in the lower subfigure denotes the $\pm {10}\%$ deviations from the Monte Carlo simulated LET. The dose profiles are shown for reference.

The LET from each irradiated OSLD was estimated from its (reference-corrected) UV/blue ratio through the LET calibration shown in Fig. 5b. The LET values were subsequently grouped according to the packages and the mean of each package with its standard error of the mean is shown in Fig. 6 along with the Monte Carlo simulated LET. The data is shown for each of the three proton fields with the depth-dose profile plotted with dotted lines for reference.

The estimated LET values are generally within the uncertainties of the the Monte Carlo simulated values, except for the low-LET measurements, and spans the $(0.6{-}6.5)\,\hbox {keV}\, \upmu \hbox {m}^{-1}$ LET range. The average absolute deviation between the measured and simulated LET was 5.5%. As the deposited dose also affects the UV/blue ratio, the accuracy of the LET calibration curve and determination can be improved using only OSLDs irradiated with a similar dose. The LET determination using only the OSLDs irradiated with 1 Gy at the two SOBP plateaus gives an average absolute deviation of $3.5\,\%$. Whilst it would be possible to irradiated all OSLDs with the same dose, in line with a previous study^18^, the accuracy of the LET determined from irradiations at different doses is comparable to the LET determination for OSLDs irradiated at equal doses.

### LET-corrected dose measurements

**Figure 7:**
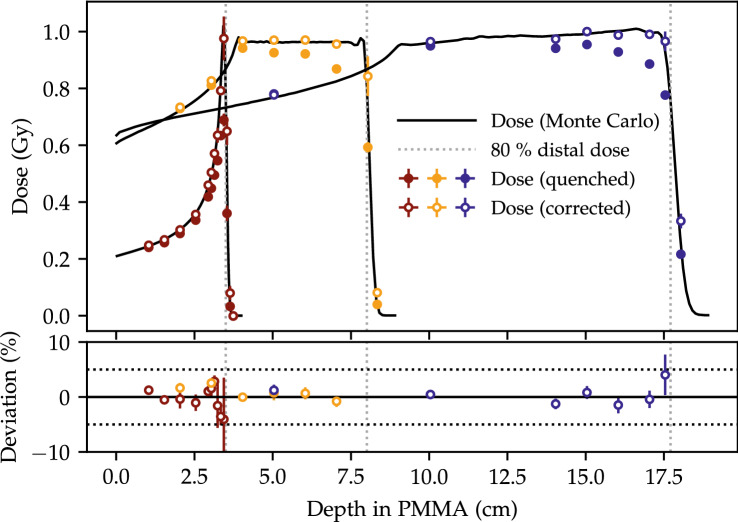
The measured quenched dose (filled markers) and corrected (open markers) for the three fields using only the emission in the blue band. The deviation of the measured LET from the simulated LET in the lower figure includes only the corrected doses below the 80 % distal dose edge (vertical, dotted lines).

The quenched dose measurements estimated from the blue emission bands, using the dose calibration in Fig. 3, are shown with filled markers in Fig. 7 for the three fields. The averaged UV/blue ratio for each package is used to look up the relative detector efficiency $\eta$ from the function plotted in Fig. 4.

Each quenched OSLD dose measurement is then corrected through the inverse of the relative detector efficiency $\eta$ and the results are shown with open markers in the figure. Only the deviations of these quenching-corrected doses relative to the dose along the central beam axes are shown in the lower figure. The quenching-corrected doses for the two SOBPs are within the uncertainties of the deposited dose except for the deviation at the Bragg peak region for the 70 MeV single energy layer $f_1$, which is attributed to a minor misalignment as it is in agreement for lower dose gradients.

## Conclusions

It is demonstrated how the use of automated OSLD corrections improve both dose and LET measurements from the entrance region to the distal edge of three proton fields. The corrections reduce the OSLD variation to a 1.2% level and the variation of the UV/blue ratio to 0.6%. The OSLD corrections and point-like detector sizes presented here enable LET measurements in steep dose and LET gradients. The measurable (fluence-averaged in water) LET is extended to the $(0.4{-}6.5)\,\hbox {keV} \,\upmu \hbox {m}^{-1}$ range for doses in the $(0.2{-}1.0)\,\hbox {Gy}$ range. For this wider dose range an average deviation of 5.5% between the measured and simulated LET is expected, whereas this can be reduced to 3.5% if the dose is kept constant.

The emission in the blue band was used for the dose measurements, although the combination of the UV and blue emission bands indicates a possibility of a quenching-free quantity. The OSLD doses were quenching-corrected using the UV/blue ratio also applied for the LET measurements and were within the ($k = 1$) uncertainties of the delivered doses for the spread-out Bragg peaks.

The automated corrections for $\text {Al}_2\text {O}_3\text {:C}$ OSLDs improve the simultaneous LET and dose measurement capabilities in proton beams, which, along with its dose-rate independence and point-like size, makes the detector suitable to support radiobiological experiments in proton beams.

## Supplementary Information

Supplementary Information.

## Data Availability

All data required to reproduce results presented in this work is accessible at: 10.17632/7b52nxhxbv.
