# Supplementary material for: Improved simultaneous LET and dose measurements in proton therapy
Source: Sci Rep. 2022 May 18;12:8262. doi: 10.1038/s41598-022-10575-4 (PMC9117334; doi:10.1038/s41598-022-10575-4)
Supplement: Supplementary file 1 — Supplementary Information. [file 41598_2022_10575_MOESM1_ESM.pdf]

A Supplementary material

A.1 Al_2O_3:C OSL emission

The UV and blue emissions for proton irradiations with 0.5 keV µm^{-1}, 1.4 keV µm^{-1}, and 9.1 keV µm^{-1}, respectively. The OSL curves in all figures were normalized to the blue emission to highlight how the UV intensity increases with increasing LET. Each curve shows the average of five OSLD readouts where the standard deviation ($k = 1$) is plotted around the curve.

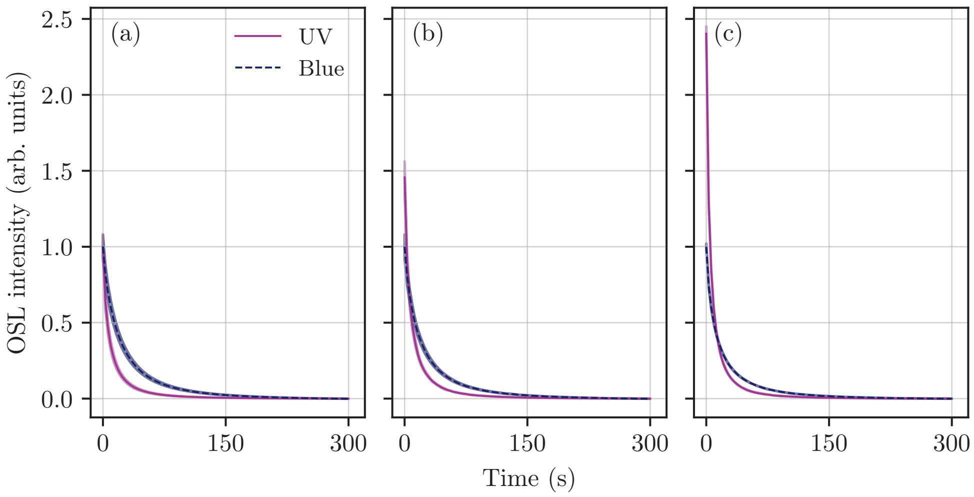

Figure A.1: The OSL signals from the UV and blue emission bands for measurements at (a) 0.5 keV µm^{-1} (b) 1.4 keV µm^{-1}, and (c) 9.1 keV µm^{-1}, where the standard deviation ($k = 1$) of the mean of the five OSL curves are shown with a shaded curves around the mean plotted with lines. The OSL signals in each figure were normalized to the intensity of the blue. The legend is representative for all three figures.

A.2 Signal fading and build-up

The OSLDs were stored for two weeks after proton irradiations before readout to reduce the effects of fading and build-up during the 50 h readout window. The behaviour of the quantities of interest as a function of storage time is shown in figure A.2. Each data point in the figure denotes the mean and the standard deviation data for the OSLDs in each package after corrections, where the function in eq. (1b) was fitted to the data series. The fading of the blue emission band in figure A.2(a) is negligible during the time the OSLDs were read out, in agreement with literature studies.[13,29,36] The build-up of the UV emission band and the UV/blue ratio was determined to vary less than 0.5 % during the readout and no build-up nor fading correction factors were applied. Nonetheless, a more stable UV emission band would enable readouts and LET estimations shortly after the irradiation as required for clinical applications. The nature of the fading or build-up for the two emission bands in Al_2O_3:C remains unclear.[14]

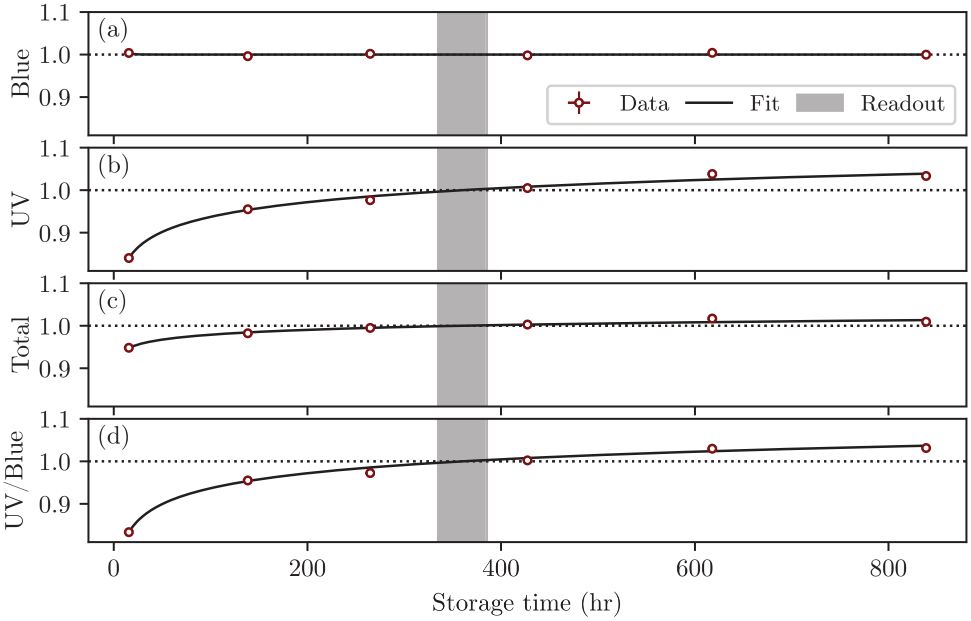

Figure A.2: Relative build-up or fading of the four quantities of interest as a function of storage time after irradiation. Total denotes the sum of the UV and blue emission bands during stimulation. Each quantity is normalized to the middle of the readout window delineated in gray.
